# Editorial

**Published:** 1846-01

**Authors:** 


### THE MEDICAL EXAMINER.

PHILADELPHIA, JAN., 1846.

The present number of the *Examiner* is the commencement of the ninth year of its existence, and the third year of our editorial labours.

A year ago, we ventured to commence a *new series* of the Journal, on a plan different from that which had previously been pursued, and to issue the numbers at periods of a month, instead of every fortnight. The design of these changes was to gain space for the insertion of articles of a more elaborate character than its former limits permitted, and to afford to its patrons a larger amount of reading matter, without additional cost to them. The editor hoped by these arrangements to be able to give increased interest to its pages, and the publishers relied upon this to obtain for the journal a circulation equal to the additional cost of its publication. We rejoice to be able to say, that these anticipations have been fully realized, and that we have now the satisfaction of addressing a number of subscribers far larger than was ever before inscribed upon its list. In this circumstance we have the best evidence of the opinion of the profession of the character of the journal, and an undoubted guarantee of its continuance.

Gratified by this unequivocal evidence of the approbation of the profession, we shall endeavour to merit its continuance by greater efforts for the promotion of all its best interests, and especially by the collection and dissemination of the improvements which from time to time occur in the various branches of medical science. To this end, we earnestly solicit the co-operation of our brethren in all parts of this widely extended country. Well written papers on medical subjects, accounts of interesting cases, and of endemic and epidemic diseases, will always be particularly acceptable.

---

EPIDEMIC SMALL-POX.

We have never known small pox to be so prevalent throughout the country as at the present time. Cities, towns and villages, every where, are infested with it to a great extent; and what is remarkable, the epidemic seems to be as mild as it is prevalent. The great majority of cases occur in persons who have undergone a degree of protection by having previously had the disease or been vaccinated, and in such, as usual, it is greatly modified—the attack consisting of more or less pain in the head and back, some nausea, fever for a day or two at the commencement, with a very sparse eruption, and *no secondary fever*. Such cases require very little treatment, recover in from three to five or six days after the first appearance of the eruption, and are followed by no disfiguration. When the disease attacks those who have not previously been vaccinated successfully, or have not had the variolous disease, it runs the ordinary course of unmitigated small pox; in some instances being discrete, and in others confluent, according to the constitution and treatment of the patient. In Philadelphia, where the disease has been quite prevalent for more than a month past, we have heard of no instance in which it has proved fatal where the subject was known to have been successfully vaccinated, and the deaths that have occurred, as far as have come to our knowledge, have been confined to such as had never been vaccinated, or in whom the proper vaccine mark had disappeared, if it had ever existed. It is a subject of astonishment and regret, that in an enlightened community like that in which we live, so much laxity and obstinacy should prevail in regard to the necessity of vaccination. In repeated instances, since the present epidemic has appeared, we have had occasion to vaccinate two and three persons in one family, mostly children or servants, who had until the time been neglected. How can it be expected that we shall be exempt for any long time from a disease so communicable, while such carelessness and stupidity prevails? A large proportion of the unprotected cases that occur in Philadelphia, are in persons who have come hither from remote or surrounding places, and it would appear that an equal degree of the carelessness to which we have referred prevails all over the country. Even in the eastern states, among a people so proverbial for their prudence, the same heedlessness prevails. According to the testimony of our brother of the Boston Medical and Surgical Journal, this would seem to be especially the case in the State of Maine.

---

NEW YORK MEDICAL INTELLIGENCER.

The publication of this periodical we learn has been discontinued—we presume from the want of sufficient patronage, certainly from no lack of ability in the Editor, or of materials to make the journal interesting.

---

TO AUTHORS AND CORRESPONDENTS.

We have received several valuable communications, which shall appear in our next number. Books, Introductory Lectures, &c., now on hand, will also be noticed.

